# Using Genipin to Immobilize Bone Morphogenetic Protein-2 on Zirconia Surface for Enhancing Cell Adhesion and Mineralization in Dental Implant Applications

**DOI:** 10.3390/polym12112639

**Published:** 2020-11-10

**Authors:** Ying-Sui Sun, Yu-An Lin, Her-Hsiung Huang

**Affiliations:** 1 School of Dental Technology, Taipei Medical University, Taipei 11031, Taiwan; yingsuisun@tmu.edu.tw; 2 Department of Dentistry, National Yang-Ming University, Taipei 11221, Taiwan; 3 Institute of Oral Biology, National Yang-Ming University, Taipei 11221, Taiwan; ukorose@gmail.com; 4 Department of Bioinformatics and Medical Engineering, Asia University, Taichung 41354, Taiwan; 5 Department of Medical Research, China Medical University Hospital, China Medical University, Taichung 40402, Taiwan; 6 Department of Stomatology, Taipei Veterans General Hospital, Taipei 11217, Taiwan; 7 Department of Education and Research, Taipei City Hospital, Taipei 10341, Taiwan

**Keywords:** natural cross-linker genipin, bone morphogenetic protein-2 (BMP-2), human bone marrow mesenchymal stem cell, cell adhesion, cell mineralization

## Abstract

Our objective in this study was to promote cell responses through the immobilization of bone morphogenetic protein-2 (BMP-2) on roughened zirconia (ZrO_2_) through using the natural cross-linker genipin in dental implant applications. Field emission scanning electron microscope, X-ray photoelectron spectroscopy, and attenuated total reflection-Fourier transform infrared spectroscopy were used to analyze the surface characterizations, including the topography, chemistry, and functional groups, respectively, of the test specimens. Human bone marrow mesenchymal stem cells (hMSCs) were used to detect cell responses (adhesion, proliferation, and mineralization). The surface characterizations analysis results revealed that genipin was effective in immobilizing BMP-2 on roughened zirconia surfaces. BMP-2 proved effective in promoting the adhesion and mineralization of hMSCs on roughened zirconia. The surface modification proposed has potential in zirconia dental implant applications.

## 1. Introduction 

Tooth loss is commonly dealt with using removable dentures or dental bridges; however, many patients are dissatisfied with their functionality and aesthetic appearance. Titanium (Ti) dental implant is highly biocompatible; nevertheless, its metallic gray color is not aesthetically pleasing and its corrosion product may induce allergic reactions in patients [1,2]. Zirconia (ZrO_2_) is a viable alternative to Ti as an implant material due to its good mechanical properties, biocompatibility, corrosion resistance, tooth-like appearance, and low plaque affinity [3]. However, bioinert zirconia surfaces tend to hinder bonding between implant and bone. Previous studies have shown that the roughening of the surface via physical treatments (such as sandblasting) improves the mechanical interlocking of the dental implant with the surrounding bone [4] and the adhesion, metabolic activity, and proliferation of human osteoblast-like cells [5]. Treatments involving the immobilization of biologically active molecules on zirconia surfaces have also been shown to enhance the bioactivity (i.e., Ca/P formation ability) [6,7]. Basically, surface modification aiming at improving the bone cell response of bioinert zirconia surfaces still needs detailed investigations [8].

Bone morphogenetic protein-2 (BMP-2) belongs to the transforming growth factor beta superfamily of proteins [9]. It is a potent osseoinductive factor involved in a variety of biological functions associated with osteogenesis and osteogenic differentiation, including the maintenance of normal bone as well as bone regeneration [9,10,11,12,13]. BMP-2 has also been shown to reduce bone resorption and improve osseointegration in dental implant applications [14]. Note, however, that a high dose of BMP-2 may induce apoptosis and cytotoxicity in periodontal ligament cells [15]. Recombinant human bone morphogenetic protein-2 (rhBMP-2) has been approved by the U.S. Food and Drug Administration (FDA) to promote the formation of bone and cartilage through induced osteoblast differentiation [16,17]. The overexpression of adenoviral-mediated BMP-2 promotes the upregulation of osteogenic gene expression, such as alkaline phosphatase (ALP) and runt-related transcription factor 2 (RUNX2), in bone marrow stem cells (BMSCs) [18]. Thus, it is interesting to know how BMP-2 positively affects the bone cell response to a bioinert zirconia surface without causing biological side effects. 

Genipin is a chemical compound extracted from the fruit of Gardenia jasminoides which acts as a natural cross-linker for proteins, collagen, gelatin, and chitosan [19]. Genipin can be used in a similar manner to glutaraldehyde, the latter of which is widely used in clinical settings to enhance the mechanical properties of biomaterials; however, genipin is superior to glutaraldehyde in terms of cell cytotoxicity, biocompatibility, mechanical properties, and resistance to enzyme degradation in vivo [20]. In traditional Chinese medicine, genipin serves as an inhibitor of uncoupled protein 2, which has been shown to promote the release of insulin in patients with diabetes mellitus type 2 [21]. The antioxidant and anti-inflammatory effects of genipin have also been shown to suppress liver apoptosis in mice [22]. Based on the abovementioned literature, in the current study we used genipin as a simple, cost-effective natural cross-linker to immobilize bioactive BMP-2 on bioinert zirconia surfaces to enhance the bone cell responses in dental implant applications.

## 2. Materials and Methods 

### 2.1. Specimen Preparation 

Biomedical-grade zirconia disks with diameters of 15 mm and thicknesses of 3 mm were used as test substrates (designated as Z). The Z specimens were sandblasted using aluminum oxide particles (size 120 µm) for 10 sec by a dental sandblaster (pressure 4 bar) at a 5 cm distance from the surface of the disk. The resulting sandblasted zirconia specimens were designated ZS. Several ZS specimens were subjected to alkaline immersion treatment in 5M of NaOH at 60 °C for 24 h to attract hydroxyl (OH) groups to the roughened surface. The same specimens were then immersed in phosphate-buffered saline (PBS) containing genipin (0.05%) at 37 °C for 24 h. Note that genipin binds to hydroxyl groups on the specimen surface via the aldehyde groups (CO). The resulting genipin-treated ZS specimens were designated ZSG. Several ZSG specimens were immersed in ddH_2_O containing BMP-2 (10 ng/mL) at 37 °C for 24 h. The N-terminals of the proteins interact with genipin to form amide bonds leading to the immobilization of BMP-2 on the roughened zirconia surface. The resulting BMP-2-immobiblized ZSG specimens were designated ZSGB. 

### 2.2. Surface Characterization 

Field emission scanning electron microscope (FESEM, JEOL Ltd., Tokyo, Japan), X-ray photoelectron spectroscopy (XPS, ULVAC-PHI, Inc., Kanagawa, Japan), and attenuated total reflection-Fourier transform infrared spectroscopy (ATR-FTIR, PerkinElmer Inc., Waltham, MA, USA) were respectively used to analyze the surface morphology, chemical elements, and functional groups of the specimens.

### 2.3. Cell Responses 

#### 2.3.1. Cell Culture 

hMSCs were cultured in Dulbecco’s Modified Eagle’s Medium-low glucose (DMEM-low glucose; Merck KGaA, Darmstadt, Germany) containing 10% fetal bovine serum (FBS; Thermo Fisher Scientific, San Jose, CA, USA), 1.5 g/L of sodium bicarbonate, and 1% penicillin/streptomycin and maintained at 37 °C under humidified air with 5% CO_2_.

#### 2.3.2. Cell Adhesion 

hMSCs were seeded at a density of 1.5 × 10^4^ cells/specimen on test specimens. Following incubation for 6 h, cells adhering to the test specimens were fixed using fixation buffer (2% glutaraldehyde + 4% paraformaldehyde) and then dehydrated in a series of ethanol (10–100%). After coating the specimens with a platinum film, the cell adhesion morphology was observed using FE-SEM.

#### 2.3.3. Cell Proliferation 

Human bone marrow mesenchymal stem cell (hMSCs) (passage 5–8) were seeded at a density of 1 × 10^4^ cells/specimen on test specimens. Following incubation periods of 1, 3, and 5 d, the cell proliferation was evaluated by adding alamarBlue^®^ reagent (Thermo Fisher Scientific, Waltham, MA, USA) to a final concentration of 10%, followed by incubation at 37 °C for 1, 3, and 5 d. The reducing environment of the cells in the alamarBlue assay was measured in terms of the conversion of resazurin (oxidized form) to resorufin (reduced form) and expressed as a percentage reduction in alamarBlue as a function of culture time. 

#### 2.3.4. Cell Mineralization 

The extracellular matrix (ECM) produces calcium deposits in the mineralization stage of osteogenic differentiation. hMSCs were seeded at a density of 5 × 10^3^ cells/specimen on test specimens, then were cultured in normal medium for 24 h and moved to an osteogenic medium containing DMED (Merck KGaA, Darmstadt, Germany) supplemented with 10^−8^ M of dexamethasone, 10 mM of ß-glycerophosphate, and 50 µg/mL of ascorbic acid. Afterwards, the osteogenic medium was changed every two days. Following incubation periods of 7 and 21 d, the cell mineralization was evaluated by adding 1% Alizarin red S in distilled water (pH 4.2) for 1 h at room temperature (RT) to detect calcium components following cell fixation using ice-cold 70% ethanol. The cells were subsequently rinsed using distilled water until the color disappeared. The stained cells were observed and imaged using a metallographic microscope. The quantification of stained cells was performed by adding 10% (*w/v*) cetylpyridinium chloride in 10 mM of sodium phosphate (pH 7.0) at RT for 1 h to extract Alizarin red S. The absorbance at 540 nm was measured using a microplate reader. A higher optical density (OD) represented a higher mineralization ability.

### 2.4. Statistical Analysis 

The results of cell proliferation and mineralization were presented as mean with standard deviation (SD). Student’s t-test was used for statistical analysis, with a p value < 0.05 as statistical significance.

## 3. Results and Discussion 

### 3.1. Surface Characterization

**Figure 1:**
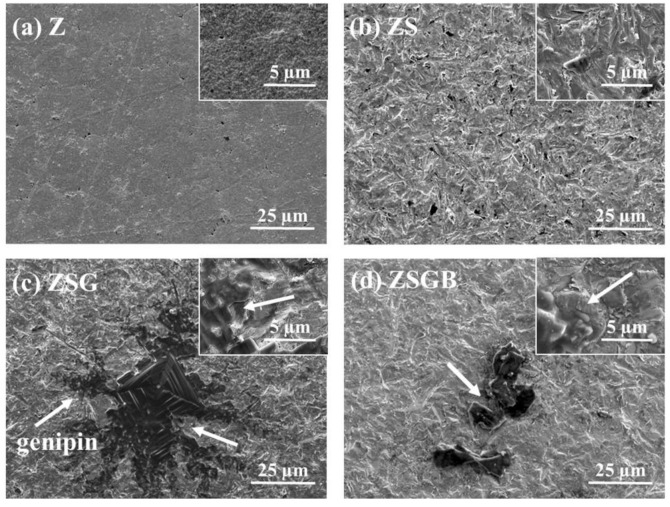
FESEM micrographs, showing the surface morphology of test specimens: (**a**) Z (untreated zirconia), (**b**) ZS (sandblasted Z), (**c**) ZSG (genipin-treated ZS), and (**d**) ZSGB (BMP-2-immobilized ZSG); the upper right corner of each figure is a partial enlarged view (arrows: genipin).

Figure 1 presents FESEM micrographs showing the surface morphology of Z, ZS, ZSG, and ZSGB. The ZSG specimens presented a snowflake-like pattern with a random distribution. The ZSGB specimens presented an irregular flake layer and the BMP-2 was mixed with the genipin. Several biomaterials have been used to mimic different biological microenvironments, such as genipin-crosslinked chitosan [23]. The immobilization of genipin to produce a biomolecular surface on Ti alloy has considerable potential for bone implant applications [24]. In this study, we immobilized the BMP-2 as an activator to improve bone cell responses, as will be discussed later.

**Figure 2:**
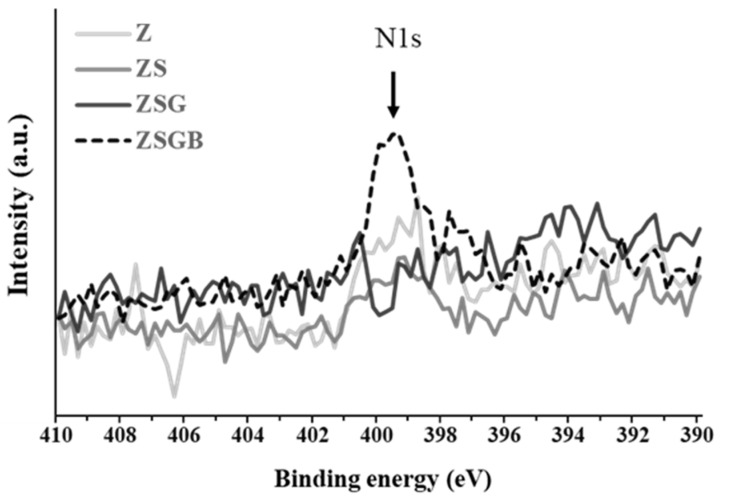
XPS analysis results, in terms of N1s (1s orbital for nitrogen) spectra, of test specimens.

**Figure 3:**
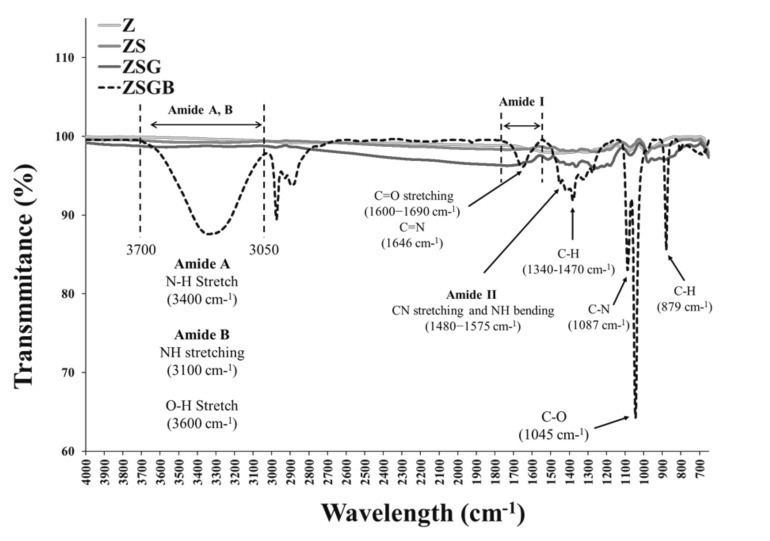
FTIR analysis results showing the function groups of test specimens.

Figure 2 presents the XPS analysis results in terms of the N1s (1s orbital for nitrogen) spectra. According to the Thermo Scientific™ data system [25], the binding energy of N1s of amine groups (-NH2) is around 399–400 eV. The N1s intensity of ZSGB (attributable to the N-terminal of BMP-2 proteins) was higher than that of ZSG, indicating the successful immobilization of BMP-2. Figure 3 presents the typical ATR-FTIR spectra of test specimens. The ZSGB specimens revealed the C=O stretching vibrations of amide I (1600–1690 cm^−1^), the C–N stretching and N–H bending vibrations of amide II (1480–1575 cm^−1^), and the N–H stretching vibrations of amide A and B (3050–3700 cm^−1^). This confirmed again the presence of BMP-2 in ZSGB specimens.

The FTIR analysis results (Figure 3) indicated the successful immobilization of BMP-2 on roughened zirconia via amide groups between BMP-2 and genipin. Recently, BMP-2 has been successfully immobilized on porous Ti surfaces though genipin to enhance the bone cell response [26]. For future application potential, BMP-2 can be immobilized on ceramics as a local delivery growth factor [27].

### 3.2. Cell Responses 

**Figure 4:**
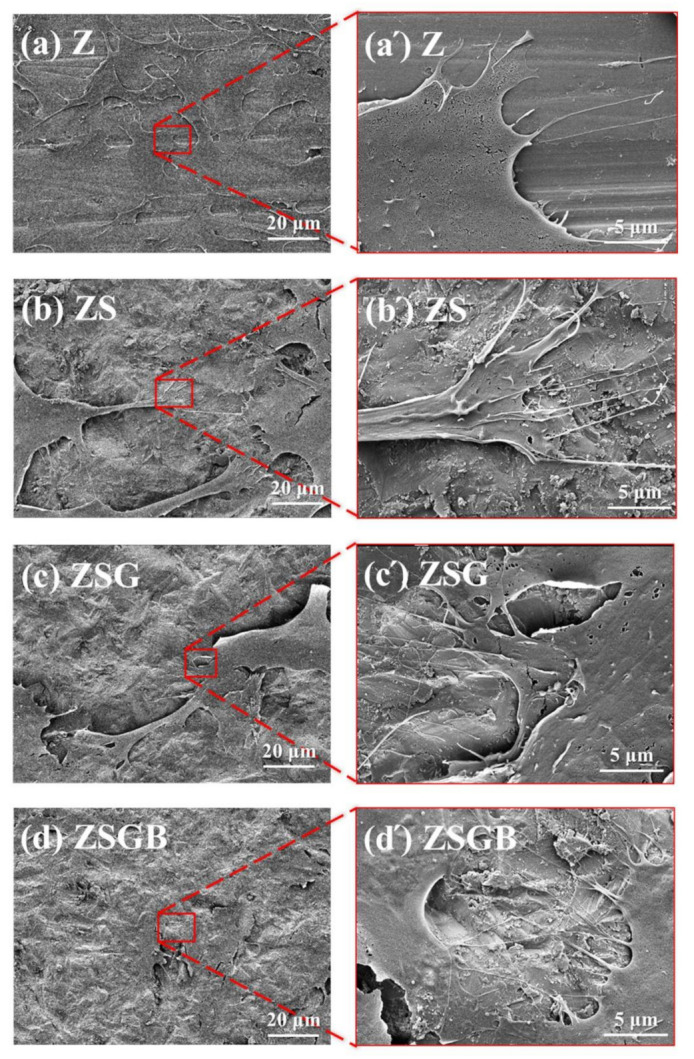
FESEM micrographs showing the cell morphology on test specimens after 6 h of incubation: (**a**) Z (untreated zirconia), (**b**) ZS (sandblasted Z), (**c**) ZSG (genipin-treated ZS), and (**d**) ZSGB (BMP-2-immobilized ZSG); the micrographs (**a’**–**d’**) are partial enlarged views of the micrographs (**a**–**d**), respectively.

Figure 4 presents the FE-SEM analysis results of hMSCs following cell incubation for 6 h. The morphology of cells on BMP-2-immoblized specimens (ZSGB group) presented better spreading (flattening) morphology and cell–cell communication (as indicated by white arrows on the right micrograph) than that observed in the other specimens. Cell adhesion includes the propagation of a thin cell membrane, followed by the spreading of cell filopodia. The flattened cell morphology indicated a good cell adhesion, representing a good biocompatibility [28]. A research demonstrated that BMP-2 significantly alters the osteoblastic cytoskeletal and ECM organization and enhances the expressions of fibronectin and specific integrin receptor subunits, which contributes to downstream cellular responses that are important for bone cell growth [29]. 

**Figure 5:**
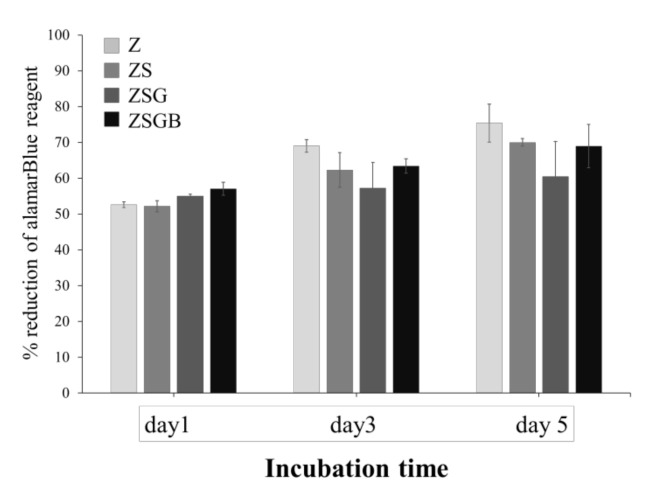
Cell proliferation of test specimens during the 5 d of incubation.

Figure 5 illustrates the cell proliferation of hMSCs on test specimens throughout the 5-day cell incubation period. After 5 d of incubation, no statistically significant differences were observed among the test specimens in terms of cell proliferation. However, the average cell proliferation in the ZSG and ZSGB specimens was lower than that in the Z and ZS, indicating that BMP-2 had no positive effect on cell proliferation at day 3 and day 5. This result may imply that the hMSCs on ZSG and ZSGB surfaces proceeded faster to the later stage of cell growth—namely, the mineralization stage of osteogenic differentiation.

**Figure 6:**
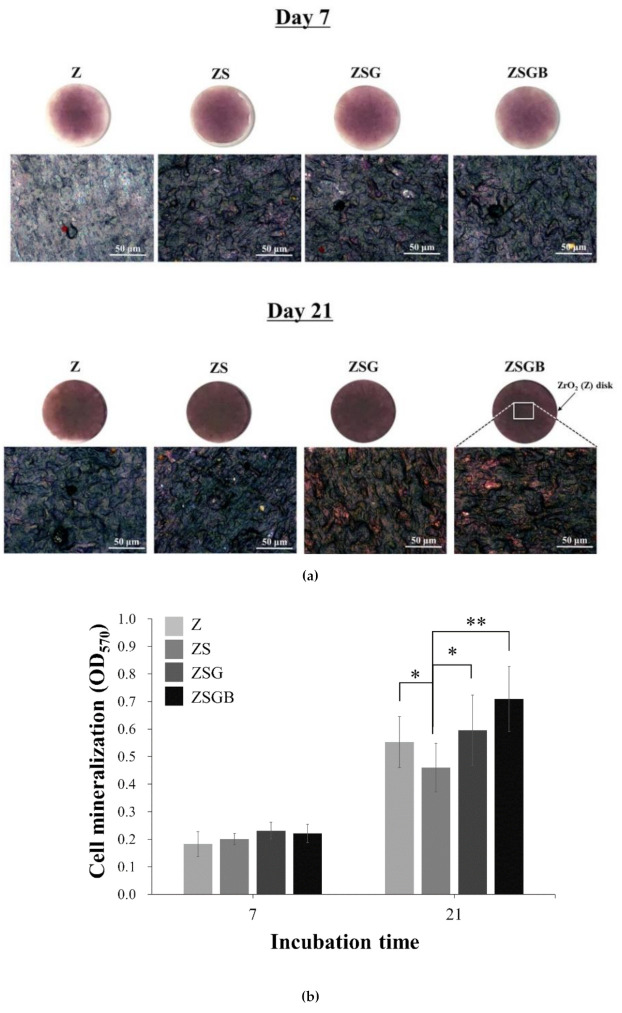
Cell mineralization analysis results, in terms of Alizarin red S staining, of the test specimens after 7 d and 21 d of incubation: (**a**) qualitative observation (upper row: zirconia disk; lower row: higher magnification of upper row); (**b**) quantitative analysis (* *p* < 0.05; ** *p* < 0.01).

Figure 6 presents the cell mineralization analysis results, in terms of the Alizarin red S staining, of the test specimens after 7 d and 21 d of incubation. Based on the (a) qualitative and (b) quantitative data, no significant differences were observed in the mineralization ability at day 7, while ZSBG had the highest mineralization ability at day 21. It has been proved that the immobilization of BMP-2 on the porous Ti surface can considerably enhance the cell mineralization and biological activity, causing the surface of biomolecules to have excellent application potential in bone implantation [26]. In this study, Figure 6 shows that the cell mineralization on the roughened zirconia (TS) surfaces is significantly improved by the immobilization of BMP-2 (at day 21). This confirmed that the lower proliferation (day 5 in Figure 5) and faster mineralization (day 21 in Figure 6) of hMSCs on ZSGB surfaces advise that the immobilization of BMP-2 on roughened zirconia surfaces enhanced the bone cell growth. According this result, we suggest that the cross-linker genipin stabilized the immobilization of BMP-2 on roughened zirconia surfaces and reduced the degradation of BMP-2, resulting in the highest mineralization ability of ZSGB at day 21. However, this still needs further investigation.

According to a previous report, sandblasting can improve the adhesion and proliferation of human osteoblast-like cells on the surface of zirconia [5]. Note, however, that the rough ZS surface treated by sandblasting had a better cell adhesion morphology in terms of cell filopodia stretching (Figure 4), but a lower cell proliferation (Figure 5) and mineralization (Figure 6) as compared with the untreated smooth Z surface. A similar result has been reported, in that the patterned silicon nanowire surface can increase cell−substrate adhesion while limiting cell−cell communication [30]. Therefore, we speculate that the strong cell–substrate adhesion on the sandblasted rough ZS surface may limit cell–cell communication, leading to a subsequent reduction in cell proliferation and mineralization. The underlying mechanism, however, needs further investigation.

In this study, we immobilized BMP-2 on a zirconia surface using genipin as cross-linker. We comparrf our study with a previous report [23] which uses genipin to crosslink the chitosan nanoparticles to obtain hydrogels and scaffolds. We believe that both BMP-2 and chitosan contain the amine groups (–NH2) and carboxylic acid groups (–COOH) that can be cross-linked with genipin. However, in this study, the zirconia substrate after alkaline immersion treatment is believed to be chemically bonded with genipin through its surface OH groups. To our best of knowledge, there is very limited information about the use of the natural cross-linker genipin to crosslink biomolecules to zirconia surfaces. It is recommended that more detailed in vitro and in vivo biological tests be performed before the proposed surface treatment technology can be used in clinical applications.

## 4. Conclusions 

This study reports a simple and cost-effective method for the immobilization of BMP-2 on a roughened zirconia surface using the natural cross-linker genipin. The natural cross-linker genipin was shown to facilitate the immobilization of BMP-2. The immobilization of BMP-2 on roughened zirconia surfaces triggered bone cell adhesion and mineralization, which was expected to accelerate the following osteogenesis. Overall, the proposed surface treatment scheme has considerable potential in zirconia dental implant applications.
